# Drought-Tolerant Bacteria and Arbuscular Mycorrhizal Fungi Mitigate the Detrimental Effects of Drought Stress Induced by Withholding Irrigation at Critical Growth Stages of Soybean (*Glycine max*, L.)

**DOI:** 10.3390/microorganisms12061123

**Published:** 2024-05-31

**Authors:** Aya Ahmed Nader, Fathi I. A. Hauka, Aida H. Afify, Ahmed M. El-Sawah

**Affiliations:** Department of Agricultural Microbiology, Faculty of Agriculture, Mansoura University, Mansoura 35516, Egypt

**Keywords:** arbuscular mycorrhizal fungi, indole acetic acid, gibberellic acid, exopolysaccharides, proline, phosphate solubilization

## Abstract

Considering current global climate change, drought stress is regarded as a major problem negatively impacting the growth of soybeans, particularly at the critical stages R3 (early pod) and R5 (seed development). Microbial inoculation is regarded as an ecologically friendly and low-cost-effective strategy for helping soybean plants withstand drought stress. The present study aimed to isolate newly drought-tolerant bacteria from native soil and evaluated their potential for producing growth-promoting substances as well as understanding how these isolated bacteria along with arbuscular mycorrhizal fungi (AMF) could mitigate drought stress in soybean plants at critical growth stages in a field experiment. In this study, 30 *Bradyrhizobium* isolates and 30 rhizobacterial isolates were isolated from the soybean nodules and rhizosphere, respectively. Polyethylene glycol (PEG) 6000 was used for evaluating their tolerance to drought, and then the production of growth promotion substances was evaluated under both without/with PEG. The most effective isolates (DTB4 and DTR30) were identified genetically using 16S rRNA gene. A field experiment was conducted to study the impact of inoculation with DTB4 and DTR30 along with AMF (*Glomus clarum*, *Funneliformis mosseae*, and *Gigaspora margarita*) on the growth and yield of drought-stressed soybeans. Our results showed that the bioinoculant applications improved the growth traits (shoot length, root length, leaf area, and dry weight), chlorophyll content, nutrient content (N, P, and K), nodulation, and yield components (pods number, seeds weight, and grain yield) of soybean plants under drought stress (*p* ≤ 0.05). Moreover, proline contents were decreased due to the bioinoculant applications under drought when compared to uninoculated treatments. As well as the count of bacteria, mycorrhizal colonization indices, and the activity of soil enzymes (dehydrogenase and phosphatase) were enhanced in the soybean rhizosphere under drought stress. This study’s findings imply that using a mixture of bioinoculants may help soybean plants withstand drought stress, particularly during critical growth stages, and that soybean growth, productivity, and soil microbial activity were improved under drought stress.

## 1. Introduction

Soybean (*Glycine max* L.) is considered the most cultivated seed legume, serving as a cost-effective protein source, and holds the distinction of being the predominant oilseed crop worldwide in terms of cultivation [1,2]. Global climate changes are expected to make alternations in water cycle dynamics, which in turn will have an impact on agricultural production in the future generally and soybean production specifically, especially given that soybean plants are relatively susceptible to drought stress conditions, particularly at certain growth stages [3]. The most critical periods for drought stress in soybeans are during the flowering stage and the period after flowering [4]. An adequate water supply is necessary for high-yielding soybean cultivation; nevertheless, plants can only endure water stress up to a certain degree (threshold level), after which the yield drops considerably [5,6]. The soybean yield is reduced by 40% under drought (40% field capacity) when compared to well-watered conditions [7]. Egypt is one of the most water-stressed countries due to the fixed allocation of Nile River water (55.5 billion m3 each year) and rapid population expansion. In general, abiotic stresses cause a variety of changes in plant growth due to their effects on the overall morphological, biochemical, and molecular mechanisms in plants [8,9,10,11,12], which create great challenges for plant scientists in applying sustainable approaches for plant adaptation to these conditions. Hence, developing cost-effective and environmentally friendly technology for soybean cultivation to withstand expected drought stress is of great importance to be used for sustainable agriculture in Egypt.

In this context, the usage of plant growth-promoting rhizobacteria (PGBR) has the potential to give alternative and sustainable solutions to challenges connected to crop productivity [13,14,15] and abiotic stress tolerance [16,17]. PGPR can enhance plant nutrient acquisition and can produce growth-promoting substances, as well as the production of certain substances and enzymes that mitigate the high oxidative stress under drought stress [18,19,20,21]. For example, the drought-tolerant *Bacillus pumilus* SH-9 strain can improve soybean growth under drought stress by promoting phosphate solubilization, the production of indole acetic acid, exopolysaccharides, and siderophore, as well as through the modulation of phytohormone gene expression, and the antioxidant profile in soybean plants [20]. In addition, rhizobia (*Bradyrhizobium japonicum*) can coexist in a symbiotic relationship with leguminous plants such as soybeans, which are crucial for promoting plant growth due to N_2_ fixation and the production of growth-promoting substances [22]. Many studies have reported the role of *Bradyrhizobium japonicum*; Jabborova et al. [23] reported that the synergistic use of *Br. japonicum* USDA-110 and *Pseudomonas putida* NUU8 improved soybean growth and modulation under drought. Moreover, Sheteiwy et al. [24] stated that the combined use of *Br. japonicum* USDA-110 and AMF could enhance the physiological and biochemical responses of soybean plants toward drought stress.

Among sustainable solutions to withstand drought stress, arbuscular mycorrhizal fungi (AMF) promote water and nutrients uptake, while also increasing plant resistance towards a biotic stress, including drought [25,26,27], salinity [28], and heavy metals [29,30]. The fungal hyphae can extend into the soil rhizosphere to absorb water and nutrients, helping plants uptake phosphorus from the soil, improving the soil structure by forming a sticky-string-bag of glomalin-containing hyphae, and improving the soil fertility [31,32]. Arbuscular mycorrhiza can form a symbiotic relationship with several plants including soybeans [27,33]; therefore, AMF inoculation techniques have been applied in field trials to improve soybeans’ growth, yield, and seed quality under drought stress. For example, Sheteiwy et al. [24] found that AMF inoculation led to enhanced growth and yield, as well as a reduction in oxidative damage induced by drought stress, and AMF inoculation diminished the inhibition of cell progress caused by drought stress and resulted in a shorter time for DNA accumulation and cycle division. Another study by Sheteiwy et al. [34] found an increase in the soluble sugars, lipids, protein, and oil contents in soybean seeds due to AMF inoculation when compared to untreated plants under drought. Furthermore, dual inoculation with AMF and selected rhizobia could be very necessary for soybeans for nutrient mobilization and water uptake when cultivated under water-limiting conditions [35]. Hence, AMF inoculation could be used as a strategy to increase the quantitative and qualitative traits of soybeans under drought stress.

Therefore, this study was conducted to isolate newly native drought-tolerant bacteria and tested their potential to produce growth-promoting substances under drought stress. Moreover, we studied the influence of inoculation with these bacteria along with AMF as natural bioinoculants on soybean growth and productivity. We hypothesized that inoculation with drought-tolerant bacteria and AMF will help soybean plants to withstand drought stress, especially at the early pod stage (R3, 50 days after sowing) and seed development stage (R5, 90 days after sowing), which are very sensitive stages to drought, because of their ability to produce growth-promoting substances, increase soil nutrient availability, and the enhance soil microbial activity for sustainable agriculture. To test this hypothesis, several morphological, physiological, biochemical, and microbiological parameters were evaluated to gain a better knowledge of the underlying mechanisms of the bioinoculants’ ability to mitigate drought stress effects on soybean plants at critical growth stages.

## 2. Materials and Methods

### 2.1. Isolation of Symbiotic Bacteria

Soybean roots were collected from five fields of soybean plants growing on the farm of the Agronomy Department at Mansoura University, Dakahlia Government, Egypt. The soil from the roots was removed by washing with tap water, and the mature pinkish nodules were collected and placed in a beaker. The nodules were then sterilized for 5 min with 95% ethanol and washed with sterile distilled water once more. In a test tube, nodules were crushed with the help of a sterile glass rod in 2 mL of sterile distilled water. A loopful was taken and spread in plates containing yeast extract mannitol agar (YEMA). Inoculated plates were incubated at 30 °C for 4 days, then the colonies were picked with an inoculating loop and the purity of each colony was checked first by streaking several successive times on the YEMA media with Congo red; after that the colonies were picked, microscopically examined, and then maintained in stock on YEM agar slopes for further studies [36].

### 2.2. Isolation of Non-Symbiotic Bacteria 

Rhizosphere soil samples were collected from different fields of soybean from the farm of the Agronomy Department at Mansoura University, Dakahlia Government, Egypt. Bacterial isolation was performed by the pour-plate method. Briefly, Aliquots of 1 mL from suitable dilutions were transferred to Petri-dishes, then nutrient agar medium (OXOID) was added and mixed thoroughly thereafter, bacterial colonies were picked after three days of incubation at 30 °C, and the colonies were picked and maintained in stock on nutrient agar slopes for further studies [18]. 

### 2.3. Screening Drought-Stress Tolerance

Bacterial isolates (Bradyrhizobia and Rhizobacteria) were grown on YEM and NB liquid media, respectively, and the media were supplemented by Polyethylene glycol (PEG) with a molecular weight of 6000 at a rate of 40 gm per liter [37]. Inoculation was carried out with 24 h-old isolates, incubated for 24 h at 30 °C, then 10 mL of each flask was taken, and the optical density (OD) was estimated by a spectrophotometer at 600 nm based on the following: less than 0.3, completely sensitive; between 0.3 and 0.39, sensitive-to-tolerant, 0.4 and 0.5, tolerant; greater than 0.5, completely tolerant [38]. 

### 2.4. Estimation of Plant Growth Promotion Traits

Bacterial isolates (1 mL of 24 h-old culture) were inoculated in 250 mL conical flasks each containing 100 mL of YEM and NB liquid media for bradyrhizobia and rhizobacteria, respectively. Incubation was performed at 30 °C on a rotary shaker operating at 150 rpm. To induce drought stress, the liquid media were supplemented by PEG 6000 at a rate of 40 gm per liter. For indole acetic acid, the media were supplemented with 0.1% tryptophan, and after 48 h of incubation, IAA was assayed calorimetrically at 530 nm in the supernatant according to the study of Ahmad et al. [39]. Gibberellic acid (GA) was determined at 760 nm using a spectrophotometer by the method mentioned by Abou-Aly et al. [40]. Exopolysaccharides (EPS) were determined after 72 h of incubation and calculated as mg/L, as described by Sharath et al. [37]. Proline was determined in the bacterial supernatant by the ninhydrin colorimetric method [41]. The ability of bacterial isolates to solubilize inorganic phosphate was performed on Pikovskaya medium [42] supplemented with tri-calcium, then soluble phosphorus in the supernatant was determined calorimetrically at 660 nm by the method of Boltz and Mellon [43], modified by Hemalatha et al. [44].

### 2.5. Identification of Bacterial Isolates

The identification of the most effective bacterial isolates was performed by Sigma Scientific Services Co. (St. Louis, MO, USA) using the 16S rRNA sequence. The acquired sequences were then compared to known sequences in the NCBI database (https://blast.ncbi.nlm.nih.gov/Blast.cgi accessed on 26 May 2024) by the BLAST algorithm. MEGA 11.0 software was used to align the 16S rRNA sequences. Bacterial accession numbers for 16S rRNA sequences were obtained from the NCBI GenBank (https://www.ncbi.nlm.nih.gov/ accessed on 26 May 2024).

### 2.6. The Field Experiment

#### 2.6.1. Bacterial Inoculant Preparation

For inoculum preparation, *Br. japonicum* PP236808 was grown on YEM medium for 5 days at 30 °C (approximately 9 × 10^7^ cfu/mL). *B. subtilis* PP250150 was grown on NB medium for 2 days at 30 °C (approximately 1.4 × 10^8^ cfu/mL). The seeds were immersed in liquid bacterial inoculants with an adhesive agent (Arabic gum, 16%) for 30 min. Then, the seeds were allowed to dry naturally in the air before being transplanted. During the second irrigation, each plant received an additional 10 mL of bacterial cultures. The inoculum mixture was made by combining equal volumes of the bacterial cultures. To ensure that the non-bacterial treatments received the same nutrients, equal volumes of autoclaved inoculum were applied.

#### 2.6.2. Mycorrhizal Inoculum

*Glomus clarum*, *Funneliformis mosseae*, and *Gigaspora margarita* spores were obtained from the Agricultural Microbiology Department, Faculty of Agriculture, Mansoura University, Egypt. AMF spores were trapped for six months on *Sorghum sudanenses* Pers. roots as a host plant. For mycorrhizal treatments, five grams of trapped soil with approximately 50 spores per gram and 0.5 g of infected Sudan grass roots (with 70% colonization) were added. The inoculum was placed 5 cm below the soil surface before sowing to facilitate the fungal colonization of the soybean roots. To ensure comparable nutritional conditions, the non-mycorrhizal treatments received the same amount of autoclaved inoculum.

#### 2.6.3. Agronomic Practices

A field experiment was carried out during the summer season of 2023 at the Farm of Agronomy Department, Mansoura University, Egypt (27.00° N, 30.00° E). Table S1 shows the meteorological data for the study region. The physicochemical and biological properties of the experimental soil are presented in Table S2. Each plot was (3 m × 3.5 m), with 70 cm between ridges and 25 cm between hills. Soybean seeds cv. Giza 111 were obtained from the Agronomy Department of Mansoura University, Egypt. Three soybean seeds were sown on a hill on one side of the ridge, and the thinning was carried out after three weeks to keep 2 plants per hill. 

The chemical fertilizers (N, P, and K) were applied at the rates of 180 kg urea (46% N), 361 kg calcium superphosphate (15.5% P_2_O_5_), and 120 kg potassium sulfate (48% K_2_O), respectively, per hectare. The treatments were applied as a strip-plot design with three replicates. The irrigation treatments were serving as the horizontal plots, while the treatments of fertilization (100% NPK, 50% NPK, *Br. japonicum* + 50% NPK, *B. subtilis* + 50% NPK, AMF + 50% NPK, *Br. japonicum* + *B. subtilis* + 50% NPK, *Br. japonicum* + AMF + 50% NPK, *B. subtilis* + AMF + 50% NPK, and Mixture + 50% NPK) were serving as the vertical plots.

Irrigation was carried out every 15 days in the control plots (CK) based on the local farmers’ irrigation practices in Mansoura, Egypt (550 mm/total growing season), by pumping from a neighboring pond and transferring to the plots via pipes. A water meter was used to measure the amount of freshwater as follows: 10% (initial stage), 25% (vegetative growth), 25% (flowering), and 35% (pod development to pod fill) of the total water requirements, and the soil was naturally dried during the maturation (5%). Meanwhile, drought stress was induced by withholding irrigation for 15 days during the early pod stage (R3, 50 days from sowing: D1) and seed development (R5, 90 days from sowing: D2), according to the study of Sheteiwy et al. [24].

### 2.7. Morpho-Physiological, Nodulation, Nutrient Concentrations, and Soil Enzyme Activities

After 120 days, three plants from each treatment were randomly chosen and taken to the laboratory, followed by the shoot length, root length, leaf area, and dry weight. SPAD-502 (Minolta Co., Ltd., Osaka, Japan) was used to determine the chlorophyll contents of the youngest fully expanded soybean leaf [24]. The number of nodules on each plant’s root was calculated and their average was expressed as the number of nodules per plant. Proline was determined in the leaves of soybeans according to the method [41]. The contents of NPK were determined in soybean leaves, nitrogen was determined using the Kjeldhal method [45], phosphorus was determined calorimetrically according to Snell and Snell [46], and potassium was determined by a flame photometer [47]. For the soil enzymes, dehydrogenase was determined according to the method of Casida et al. [48] and phosphatase was determined according to Tabatabai et al. [49].

### 2.8. Microbiological Analysis

After 120 days, soybean roots were stained with 0.05% trypan blue [50]. The levels of mycorrhizal colonization (F%: Frequency of root colonization, M%: intensity of cortical colonization, and A%: arbuscule abundance in the root) were estimated using Mycocalc software (https://www2.dijon.inrae.fr/mychintec/Mycocalc-prg/download.html accessed on 26 May 2024) according to the method of Trouvelot [51]. The total bacterial count and phosphate-dissolving bacteria were counted in the soybean rhizosphere after 120 days on nutrient agar medium [52] and Pikovskaya medium [42], respectively.

### 2.9. Yield Traits

The plants were harvested at the end of the mature stage (1st of November) to determine the yield traits (number of pods/plant, 100 seeds weight, and grain yield per hectare).

### 2.10. Statistical Analysis

Data were expressed as the means ± standard deviation (SD). The data were analyzed by analysis of variance technique using SPSS v27.0 (SPSS, Inc., Chicago, IL, USA). The mean values were separated using Duncan’s multiple range test at a 0.05 level. The interaction between drought stress and fertilization was evaluated with two-way ANOVA using the software of CoStat, version 6.303 (CoHort, Monterey, CA, USA) and the asterisks indicate significant differences: * *p* < 0.05, ** *p* < 0.001, *** *p* < 0.0001 among the studied factors. Principal component analysis (PCA) and a heatmap of correlation were performed using the RStudio (PBC, Boston, MA, USA). Pearson’s correlation coefficient was performed by Origin Pro software, version 2021 (OriginLab Corporation, Northampton, MA, USA).

## 3. Results

### 3.1. Isolation, Screening, and Identification of Drought-Tolerant Bacteria

#### 3.1.1. Bradyrhizobium

A total of 30 different *Bradyrhizobium* isolates were isolated from soybean nodules on YEM agar. The results in Table 1 show the growth of *Bradyrhizobium* isolates on YEM media supplemented by PEG 6000 at the rate of 40 g/L. The results show that all the isolates can grow under drought stress conditions, but with different efficiencies. We selected isolates number 1, 2, 3, 4, 5, 6, 7, 13, 14, and 15 which gave an OD greater than 0.5, which indicates that they are completely tolerant to drought stress.

**Figure 1:**
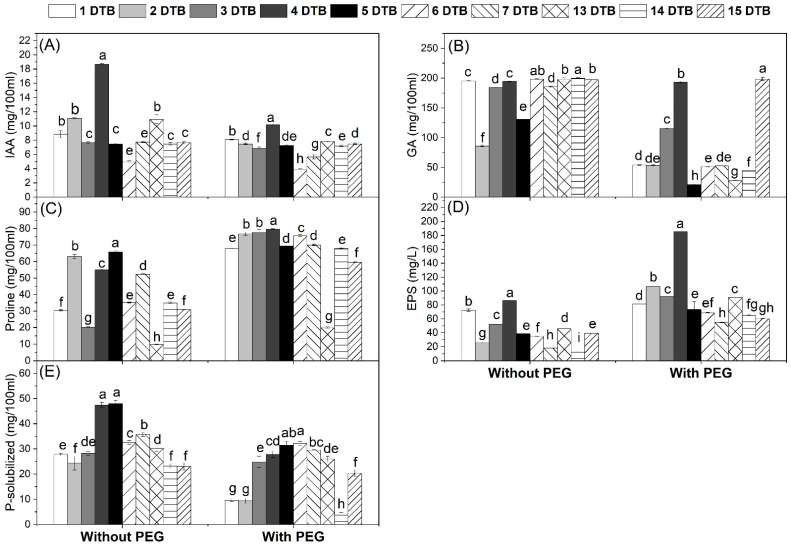
Plant growth promotion traits of drought-tolerant *Bradyrhizobium* isolates without/with polyethylene glycol 6000 (PEG). Data are means ± SD (n = 3); different letters within the same group indicate significant differences between means according to Duncan’s multiple-range test at *p* ≤ 0.05. IAA, Indole acetic acid; GA, Gibberellic acid; EPS, Exopolysaccharides. (**A**) IAA, (**B**) GA, (**C**) Proline, (**D**) EPS, and (**E**) P-solubilized.

The most tolerant ten isolates were screened for the production of IAA, GA, proline, EPS, and P-solubilization without/with PEG. The results in Figure 1A showed that all the tested isolates gave more IAA amounts under unstressed conditions when compared to drought-stressed conditions. However, the maximum IAA production under stressed conditions was recorded for isolate DTB4 (10.19 mg/100 mL) followed by isolate DTB1 (8.07 mg/100 mL), while isolate DTB6 showed the lowest amount (3.81 mg/100 mL). The same trend was observed in GA production which decreased under stressed conditions (Figure 1B). The maximum GA production under stressed conditions was recorded for isolate DTB15 (198.52 mg/100 mL) followed by isolate DTB4 (193.02 mg/100 mL), while isolate DTB5 showed the lowest amount (21.20 mg/100 mL). Regarding the production of proline, there was an increase in proline production under stressed conditions when compared to the unstressed conditions (Figure 1C). Maximum proline production under stressed conditions was recorded for isolate DTB4 (79.68 mg/100 mL) followed by the isolates DTB3 and DTB2 (77.62 and 76.70 mg/100 mL), while isolate DTB13 showed the lowest amount (20.0 mg/100 mL). Also, exopolysaccharides production showed an increase under stressed conditions (Figure 1D). The maximum exopolysaccharides production under stressed conditions was recorded for isolate DTB4 (185.31 mg/100 mL) followed by isolate DTB2 (106.23 mg/100 mL), while isolate DTB7 showed the lowest amount (55.07 mg/100 mL). For phosphate solubilization, the highest P-solubilized amounts were recorded under unstressed conditions, while the amounts decreased under stressed conditions (Figure 1E). Maximum P-solubilized under stressed conditions was recorded for isolates DTB6 and DTB5 (32.23 and 31.41 mg/100 mL) followed by isolate DTB7 (29.57 mg/100 mL), while isolates DTB2 and DTB1 showed the lowest amounts (9.37 and 9.65 mg/100 mL).

#### 3.1.2. Rhizobacteria

Thirteen different rhizobacterial isolates were isolated from the soybean rhizosphere on nutrient agar. The results in Table 1 show the growth of rhizobacteria on NA media supplemented by PEG 6000 at the rate of 40 g/L. The results show that all the isolates can grow under drought stress conditions, but at varying rates. We selected isolate number 1, 2, 3, 17, 18, 20, 23, 24, 29, and 30, which gave an OD greater than 0.3 and range from moderately tolerant to completely tolerant to drought stress. 

**Figure 2:**
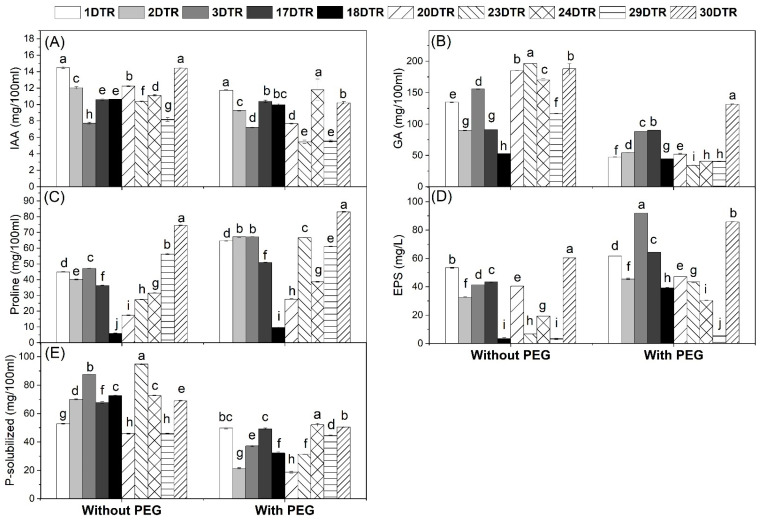
Plant growth promotion traits of drought-tolerant Rhizobacteria isolates without/with polyethylene glycol 6000 (PEG). Data are means ± SD (n = 3); different letters within the same group indicate significant differences between means according to Duncan’s multiple-range test at *p* ≤ 0.05. IAA, Indole acetic acid; GA, Gibberellic acid; EPS, Exopolysaccharides. (**A**) IAA, (**B**) GA, (**C**) Proline, (**D**) EPS, and (**E**) P-solubilized.

The most tolerant ten isolates were screened for the production of IAA, GA, proline, EPS, and P-solubilization without/with PEG. The results in Figure 2A showed that all the tested isolates gave more IAA amounts under unstressed conditions when compared to drought-stressed conditions. However, the maximum IAA production under stressed conditions was recorded for isolates DTR24 and DTR1 (11.79 and 11.74 mg/100 mL) followed by isolates DTB17 and DTR30 (10.39 and 10.22 mg/100 mL), while isolates DTB23 and DTB29 showed the lowest amount (5.54 and 5.46 mg/100 mL). Similarly, GA production was higher in unstressed than stressed conditions (Figure 2B). The maximum GA production under stressed conditions was recorded for isolate DTR30 (131.58 mg/100 mL) followed by isolate DTB17 (90 mg/100 mL), while isolate DTB23 showed the lowest amount (34.13 mg/100 mL). On the other side, stressed conditions resulted in a higher production of proline and EPS than unstressed conditions (Figure 2C,D). For proline, the maximum production under stressed conditions was recorded for isolate DTR30 (83.05 mg/100 mL) followed by isolates DTB2 and DTB3 (67.20 and 67.12 mg/100 mL), while isolate DTB18 showed the lowest amount 9.58 mg/100 mL). Maximum EPS production under stressed conditions was recorded for isolate DTR3 (91.93 mg/L) followed by isolate DTB30 (85.73 mg/L), while isolate DTB29 showed the lowest amount (5.33 mg/100 mL). For phosphate solubilization, the highest P-solubilized amounts were recorded under unstressed conditions, while the amounts decreased under stressed conditions (Figure 2E). The maximum P-solubilized amount under stressed conditions was recorded for isolate DTR24 (52.07 mg/100 mL) followed by isolate DTB30 (50.59 mg/L), while isolate DTB20 showed the lowest amount (18.65 mg/L).

#### 3.1.3. Identification of the Most Potent Isolates

The isolates DTB4 and DTR 30 were selected for identification as they could produce high amounts of plant growth promotion traits under both unstressed and stressed conditions, as mentioned previously. The isolates were identified as *Bradyrhizobium japonicum* and *Bacillus subtilis*, respectively, according to the NCBI database. Accession numbers (PP236808 and PP250150) were deposited for the two isolates in NCBI GenBank, respectively (Figure S1). 

### 3.2. Bacterial Count, Mycorrhizal Colonization, and Enzyme Activities under Drought Stress

Total bacterial counts and phosphate dissolver counts in the soybean rhizosphere are presented in Table 2. The highest number of bacterial counts (total counts and P-dissolvers) was observed in the rhizosphere of soybean plants (D1, D2, and CK), respectively. As well as this, it was also observed that the bioinoculant applications led to a significant increase in bacterial counts (total counts and P-dissolvers) when compared to non-treated treatments.

The levels of AMF colonization (F%: the frequency of root colonization, M%: the intensity of cortical colonization, and A%: the arbuscular frequency in roots) were estimated in soybean roots and are presented in Table 2. Regardless of drought stress, the data revealed that the frequency of root colonization was increased with the combined applications with AMF when compared to the single application, and the highest values were observed in the mixture treatment. However, the single AMF treatment resulted in an increase in the intensity of the cortical colonization and arbuscular frequency in soybean roots when compared to the combinations with AMF. On the other side, drought stress (D1 and D2) significantly reduced mycorrhizal indices when compared to the control treatments (CK). However, the mycorrhizal indices in D2 were higher when compared to D1 (Table 2). There was no mycorrhizal colonization in non-mycorrhizal treatments.

**Figure 3:**
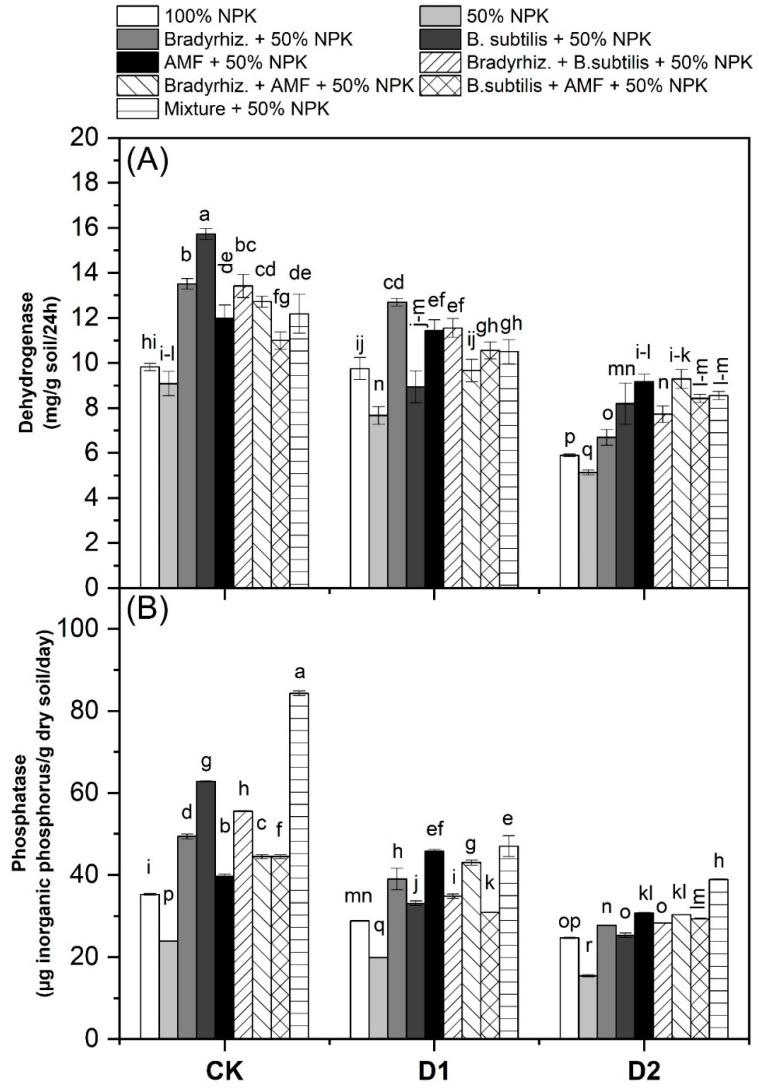
Effect of bioinoculants and their combinations on dehydrogenase and phosphatase activities in the rhizosphere of soybean plants under drought stress. Data are means ± SD (n = 3); different letters within the same column indicate significant differences between means according to Duncan’s multiple-range test at *p* ≤ 0.05. CK, Check; D1, withholding irrigation at R3 stage; D2, withholding irrigation at R5 stage; AMF, Arbuscular mycorrhizal fungi. (**A**) Dehydrogenase, (**B**) Phosphatase.

The results regarding the dehydrogenase and phosphatase activities in the rhizosphere of soybean plants are presented in Figure 3. The results revealed that the activities of dehydrogenase and phosphatase were decreased significantly due to drought stress when compared to the control treatment (CK), and this decrease was more pronounced in D2 when compared to D1 (Figure 3A,B). Regardless of drought stress, inoculation with bioinoculants increased the activities of dehydrogenase and phosphatase in the soybean rhizosphere. Dehydrogenase activity was improved with the application of *Br. japonicum* by 23.13 and 33.47%, *B. subtilis* by 23.04 and 33.39%, AMF by 22.40 and 32.84%, *Br. japonicum* + *B. subtilis* by 22.66 and 33.06%, *Br. japonicum* + AMF by 20.10 and 30.84%, *B. subtilis* + AMF by 15.67 and 27.01%, and Mixture treatment by 19.05 and 29.93% as compared with 100% NPK and 50% NPK, respectively. Phosphatase activity was improved with the application of *Br. japonicum* by 23.55 and 49.00%, *B. subtilis* by 11.57 and 41.00%, AMF by 36.29 and 57.49%, *Br. japonicum* + *B. subtilis* by 13.62 and 42.37%, *Br. japonicum* + AMF by 31.13 and 54.05%, *B. subtilis* + AMF by 15.21 and 43.43%, and Mixture treatment by 47.83 and 65.19% as compared with 100% NPK and 50% NPK, respectively. These findings demonstrated that inoculation with such bioinoculants resulted in an increase in mycorrhizal colonization as well as bacterial counts and enzyme activity in the soybean rhizosphere under both well-watered and drought stress.

### 3.3. Morphological Traits of Soybean Plants under Drought Stress

The mean data on the morphological traits of soybean plants as affected by the bioinoculant applications under drought stress are presented in Table 3. Soybean plants (CK) have the highest shoot length, root length, leaf area, and dry weight when compared to soybean plants (D1) and (D2). However, (D1) soybean plants had the lowest values of shoot length, root length, leaf area, and dry weight when compared to (D2) and (CK) soybean plants (Table 3). Regardless of drought stress, bioinoculant applications improved all morphological traits when compared to untreated soybean plants. The lowest values of the shoot length, root length, leaf area, and dry weight were found in (D1) soybean plants that were not treated with bioinoculants. The shoot length was improved with the applications of *Br. japonicum* by 2.62 and 18.45%, *B. subtilis* by 4.57 and 20.08%, AMF by 11.82 and 26.15%, *Br. japonicum* + *B. subtilis* by 5.39 and 20.76%, *Br. japonicum* + AMF by 12.42 and 26.66%, *B. subtilis* + AMF by 18.88 and 32.06%, and Mixture treatment by 19.36 and 32.47% as compared with 100% NPK and 50% NPK, respectively. The root length was improved with the applications of *Br. japonicum* by 5.45 and 33.38%, *B. subtilis* by 9.21 and 36.03%, AMF by 13.08 and 38.75%, *Br. japonicum* + *B. subtilis* by 8.06 and 35.22%, *Br. japonicum* + AMF by 18.56 and 52.61%, *B. subtilis* + AMF by 41.95 and 59.09%, and Mixture treatment by 35.89 and 54.82% as compared with 100% NPK and 50% NPK, respectively. The leaf area was improved with the applications of *Br. japonicum* by 21.05 and 51.33%, *B. subtilis* by 5.34 and 41.64%, AMF by 44.12 and 65.55%, *Br. japonicum* + *B. subtilis* by 1.32 and 39.16%, *Br. japonicum* + AMF by 3.74 and 40.66%, *B. subtilis* + AMF by 41.88 and 64.17%, and Mixture treatment by 44.32 and 65.68% as compared with 100% NPK and 50% NPK, respectively. The dry weight was improved with the applications of *Br. japonicum* by 4.22 and 18.29%, *B. subtilis* by 0.36 and 14.99%, AMF by 19.85 and 31.62%, *Br. japonicum* + *B. subtilis* by 1.57 and 16.03%, *Br. japonicum* + AMF by 11.26 and 24.29%, *B. subtilis* + AMF by 10.46 and 23.61%, and Mixture treatment by 21.82 and 33.3% as compared with 100% NPK and 50% NPK, respectively. These results indicate that the bioinoculant applications could enhance the morphological traits of soybean plants under withholding water at critical growth stages.

### 3.4. Chlorophyll and Proline Contents in Soybean Leaves under Drought Stress

**Figure 4:**
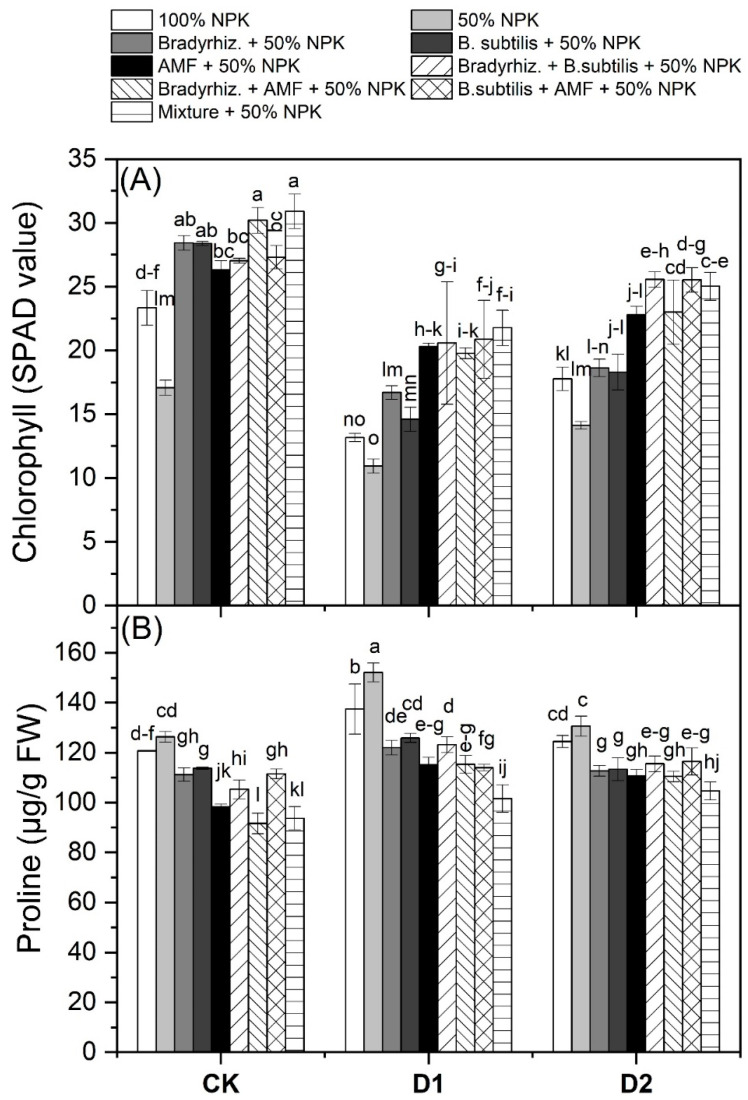
Effect of bioinoculants and their combinations on chlorophyll and proline contents of soybean leaves under drought stress. Data are means ± SD (n = 3); different letters within the same column indicate significant differences between means according to Duncan’s multiple-range test at *p* ≤ 0.05. CK, Check; D1, withholding irrigation at R3 stage; D2, withholding irrigation at R5 stage; AMF, Arbuscular mycorrhizal fungi. (**A**) Chlorophyll, (**B**) Proline.

The mean data on the chlorophyll and proline contents in soybean leaves as affected by the bioinoculant applications under drought stress are presented in Figure 4. Chlorophyll (SPAD) was improved with the applications of *Br. japonicum* by 14.88 and 33.92%, *B. subtilis* by 11.42 and 31.23%, AMF by 21.80 and 39.29%, *Br. japonicum* + *B. subtilis* by 25.83 and 42.42%, *Br. japonicum* + AMF by 25.62 and 42.26%, *B. subtilis* + AMF by 26.35 and 42.83%, and Mixture treatment by 30.15 and 45.77% as compared with 100% NPK and 50% NPK, respectively. On the other side, the proline contents were decreased by the application of *Br. japonicum* by 9.62 and 15.47%, *B. subtilis* by 7.75 and 13.72%, AMF by 15.25 and 20.73%, *Br. japonicum* + *B. subtilis* by 10.11 and 15.93%, *Br. japonicum* + AMF by 17.04 and 22.41%, *B. subtilis* + AMF by 10.64 and 16.43%, and Mixture treatment by 21.61 and 26.68% as compared with 100% NPK and 50% NPK, respectively. The obtained results indicate that the bioinoculant applications could enhance the chlorophyll contents while decreasing the proline contents in the leaves of soybean plants that are being withheld from water at critical growth stages.

### 3.5. Nutrient Contents in Soybean Leaves under Drought Stress

**Figure 5:**
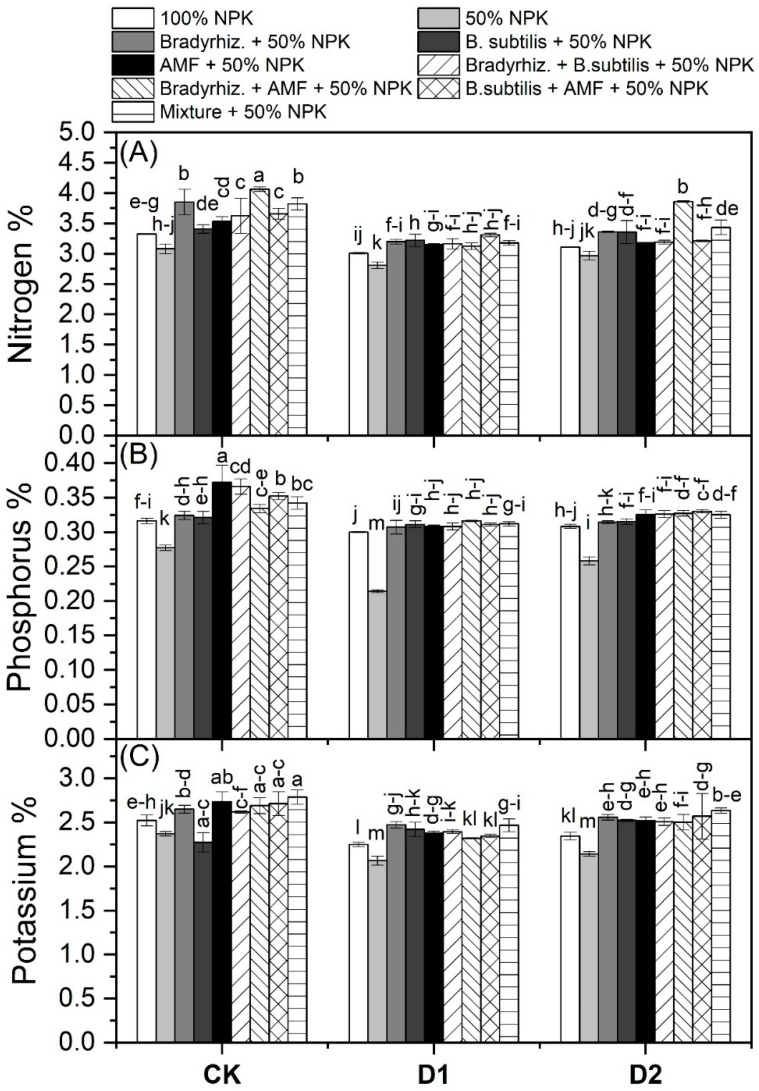
Effect of bioinoculants and their combinations on NPK contents of soybean leaves under drought stress. Data are means ± SD (n = 3); different letters within the same column indicate significant differences between means according to Duncan’s multiple-range test at *p* ≤ 0.05. CK, Check; D1, withholding irrigation at R3 stage; D2, withholding irrigation at R5 stage; AMF, Arbuscular mycorrhizal fungi. (**A**) Nitrogen, (**B**) Nitrogen, and (**C**) Potassium.

The mean data on the N, P, and K contents of soybean plants, as affected by the bioinoculant applications under drought stress, are presented in Figure 5. The data revealed that the nitrogen% was improved with the applications of *Br. japonicum* by 9.23 and 14.81%, *B. subtilis* by 5.47 and 11.28%, AMF by 4.29 and 10.17%, *Br. japonicum* + *B. subtilis* by 5.4 and 11.21%, *Br. japonicum* + AMF by 14.54 and 19.79%, *B. subtilis* + AMF by 5.25 and 11.07%, and Mixture treatment by 9.86 and 15.4% as compared with 100% NPK and 50% NPK, respectively. The phosphorus% was improved with the applications of *Br. japonicum* by 2.22 and 20.74%, *B. subtilis* by 2.42 and 20.90%, AMF by 8.05 and 25.47%, *Br. japonicum* + *B. subtilis* by 4.74 and 22.78%, *Br. japonicum* + AMF by 5.42 and 23.33%, *B. subtilis* + AMF by 6.94 and 24.572%, and Mixture treatment by 5.61 and 23.49% as compared with 100% NPK and 50% NPK, respectively. Potassium% was improved with the applications of *Br. japonicum* by 7.38 and 14.38%, *B. subtilis* by 7.30 and 14.30%, AMF by 6.82 and 13.86%, *Br. japonicum* + *B. subtilis* by 5.41 and 12.55%, *Br. japonicum* + AMF by 5.28 and 12.44%, *B. subtilis* + AMF by 6.76 and 13.8%, and Mixture treatment by 9.85 and 16.66% as compared with 100% NPK and 50% NPK, respectively.

### 3.6. Nodulation and Yield Traits of Soybean Plants under Drought Stress

The mean data on nodulation and the yield traits of soybean plants as affected by the bioinoculant applications under drought stress are presented in Table 4. The nodules/plant were improved with the applications of *Br. japonicum* by 52.83 and 64.75%, *B. subtilis* by 6.15 and 29.87%, AMF by 7.13 and 30.60%, *Br. japonicum* + *B. subtilis* by 48.27 and 61.35%, *Br. japonicum* + AMF by 43.10 and 57.49%, *B. subtilis* + AMF by 17.26 and 38.17%, and Mixture treatment by 49.71 and 62.42% as compared with 100% NPK and 50% NPK, respectively. The pods/plant were improved with the applications of *Br. japonicum* by 1.91 and 20.92%, *B. subtilis* by 20.14 and 35.61%, AMF by 5.98 and 24.19%, *Br. japonicum* + *B. subtilis* by 10.51 and 27.85%, *Br. japonicum* + AMF by 16.38 and 32.58%, *B. subtilis* + AMF by 11.87 and 28.94%, and Mixture treatment by 24.43 and 39.07% as compared with 100% NPK and 50% NPK, respectively. The seeds’ weight was improved with the applications of *Br. japonicum* by 2.07 and 23.88%, *B. subtilis* by 0.98 and 21.02%, AMF by 7.6 and 28.19%, *Br. japonicum* + *B. subtilis* by 0.48 and 22.65%, *Br. japonicum* + AMF by 3.23 and 24.79%, *B. subtilis* + AMF by 2.29 and 24.05%, and Mixture treatment by 8.17 and 28.63% as compared with 100% NPK and 50% NPK, respectively. The gain yield was improved with the applications of *Br. japonicum* by 13.48 and 44.24%, *B. subtilis* by 0.61 and 35.95%, AMF by 1.43 and 36.47%, *Br. japonicum* + *B. subtilis* by 1.02 and 36.21%, *Br. japonicum* + AMF by 15.31 and 45.42%, *B. subtilis* + AMF by 12.54 and 43.63%, and Mixture treatment by 20.23 and 48.59% as compared with 100% NPK and 50% NPK, respectively.

### 3.7. Assessment of Bioinoculants and Their Combinations Effects by Principal Component Analysis, Heatmap of Correlation, and Pearson’s Correlation Analysis under Drought Stress

**Figure 6:**
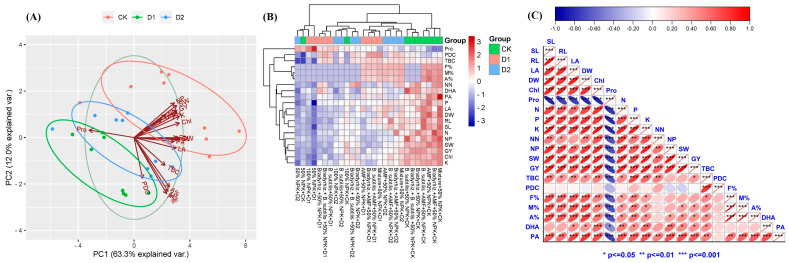
Effect of bioinoculants and their combinations by multivariate statistical analysis based on soybean plant growth attributes, physiological responses, nutrient contents, bacterial count, mycorrhizal colonization, and soil enzyme activities. (**A**) Principal component analysis (PCA). (**B**) Heatmap of correlation. (**C**) Pearson correlation analysis (SL = Shoot length, RL = Root length, LA = Leaf area, DW = Dry weight, Chl = Chlorophyll content, Pro = Proline content, N = Nitrogen, P = Phosphorus, K = Potassium, NN = Number of nodules/plant, NP = Number of Pods/plant, SW = 100 seeds’ weight, GY = Grain yield, TBC, Total bacterial count, PDC = Phosphate dissolvers count, F% = Frequency of root colonization, M% = Intensity of cortical colonization, A% = Arbuscule frequency in roots, DHA = Dehydrogenase activity, PA = Phosphatase activity).

All mean values of soybean plant growth attributes, physiological responses, nutrient contents, bacterial count, mycorrhizal colonization, soil enzyme activities, and yield attributes were analyzed by principal component analysis, PCA (Figure 6A). The PCA of the studied traits showed that two components, PC1 (contributing 63.30%) and PC2 (recording 12.00% from the entire dataset), collectively ascribed 75.30% for data variability. Moreover, a strong connection was found between the variables (growth parameters, physiological responses, nutrient contents, microbiological indices, and yield components) and the drought-stressed soybean plants treated with bioinoculants (Figure 6A,B). Furthermore, there were positive correlations between the soybean growth traits, physiological traits, nutrient contents, nodulation, yield components, and microbiological indices (Figure 6C). On the other hand, there was no significant correlation between the number of nodules (NN) and the leaf area (LA). Moreover, the total bacterial counts (TBC) did not correlate with the number of Pods/plant (NP) and 100 seeds’ weight (SW). Furthermore, the counts of phosphate dissolving bacteria (PDC) did not show any correlation with any of the parameters studied instead of the total bacterial counts (TBC). In addition, the frequency of root colonization (F%) did not show any correlation with the number of nodules/plant (NN), number of Pods/plant (NP), 100 seeds’ weight (SW), grain yield (GY), and dehydrogenase activity (DHA). Also, the intensity of cortical colonization (M%) did not show any correlation with the number of nodules/plant (NN), number of Pods/plant (NP), and dehydrogenase activity (DHA). As well as this, the arbuscular frequency in roots (A%) did not show any correlation with the number of nodules/plant (NN), the counts of phosphate dissolving bacteria (PDC), or dehydrogenase activity (DHA). Additionally, dehydrogenase activity (DHA) did not show any correlation with the shoot length (SL), root length (RL), the counts of phosphate dissolving bacteria (PDC), and mycorrhizal indices (F%, M%, and A%). Finally, phosphatase activity (PA) did not show any correlation with the counts of phosphate-dissolving bacteria (PDC).

## 4. Discussion

### 4.1. Isolated Drought-Tolerant Bacteria Showed Higher Activity to Produce Plant Growth Substances

In this study, we isolated a total of 30 *Bradyrhizobium* isolates and 30 rhizobacterial isolates that were isolated from soybean nodules and rhizosphere, respectively. All these isolates showed an ability to grow under drought-stress conditions (Table 1). The 10 most tolerant isolates from each group were screened for the estimation of the production of IAA, GA, proline, EPS, and P-solubilization without/with PEG. Our results showed that all isolates could produce these substances under both conditions. Under the drought stress conditions, IAA, GA, and P-solubilized were decreased; however, proline and EPS production were increased (Figure 1 and Figure 2). The isolates DTB 4 and DTR 30 were selected for their ability to produce high amounts of plant growth promotion traits under both unstressed and stressed conditions and these isolates were identified as *Bradyrhizobium japonicum* PP236808 and *Bacillus subtilis* PP250150, respectively (Figure S1). These findings were consistent with those of Ashry et al. [18] who found that under drought stress, various bacterial isolates produced more proline and EPS and less IAA and GA. Also, Ali et al. [19] found a higher level of EPS under stressed conditions than under non-stressed ones. This increase may indicate that proline and EPS production in bacteria occurs as a response to stress. The activity of the isolated bacteria could help soybean plants to withstand drought stress conditions. 

### 4.2. Drought-Tolerant Bacteria and AMF Improve Bacterial Counts, Mycorrhizal Colonization, and Enzyme Activities

In the current study, we observed an increase in bacterial counts due to bioinoculant applications when compared to non-treated treatments, and this increase was more pronounced in D2 treatments (Table 2). This could be linked to soil drying and rewriting, which increases the viability of nutrients and organic matter in the rhizosphere while also enhancing the soil microbial activity for decomposing organic matter as well as under drought stress where bacteria may enter a dormant stage, allowing them to quickly degrade organic materials upon rewetting [24,53]. The Pearson correlation analysis showed that the counts of phosphate-dissolving bacteria did not show any correlation with any of the parameters studied instead of the total bacterial counts (Figure 6B). This may be due to their indirect effect on plant traits through the availability of soluble phosphorus for plant uptake, and this was more pronounced in the phosphorus content in the leaves of soybeans due to bioinoculant applications (Figure 5B). Drought stress reduced mycorrhizal colonization levels (F%, M%, and A%), but the mycorrhizal species used were still able to colonize soybean roots while the mycorrhizal indices in D2 were higher when compared to D1. The highest values of mycorrhizal colonization were observed in the mixture treatment (Table 2). These results show that the inoculated AMF species could contribute to helping soybean plants absorb more water and nutrients, particularly phosphorus, from soil under drought stress by modifying the root architecture. Among the soil enzymes, soil dehydrogenase and phosphatase are direct indicators of soil microbial activity [54]. Dehydrogenase enzyme activity indicates the viability of microorganisms involved in microbial reactions in the soil, while the soil phosphatase enzyme is necessary in the mineralization of organic phosphorus. Our results showed that the activities of soil enzymes (dehydrogenase and phosphatase) were enhanced significantly in soybean rhizosphere due to bioinoculants application under drought stress (Figure 3). Also, the Pearson correlation analysis showed that the total bacterial counts were correlated with the activities of dehydrogenase and phosphatase in the rhizosphere (Figure 6C). These results indicate that the inoculation with bioinoculants resulted in high microbial activity in the soybean rhizosphere which may help soybean plants to withstand drought stress. Likewise, several studies observed an increase in the activities of soil key enzymes (e.g., dehydrogenase and phosphatase) due to the application of bioinoculants [24,54,55].

### 4.3. Drought-Tolerant Bacteria and AMF Improve Morpho-Physiological Traits of Soybean Plants

In the present study, the morpho-physiological traits of soybean have been negatively affected by D1 and D2 significantly; however, soybean growth (the shoot length, root length, leaf area, and dry weight) was improved by bioinoculants treatments under both well-watered and drought stress conditions (Table 3). This might be due to the ability of drought-tolerant bacteria to produce growth-promoting substances such as IAA, GA, proline, EPS, and P-solubilized (Figure 1 and Figure 2), which could explain such improvement in soybean growth. Moreover, AMF symbiosis improves plant growth by increasing the availability of water by their hyphae and nutrient concentration particularly phosphorus in plant tissues under drought. These results are consistent with recent findings, reporting that the use of bioinoculants (*Bradyrhizobium japonicum* and AMF) significantly increased the growth of soybeans [24]. As well as this, Sheteiwy et al. [34] found an improvement in soybean growth affected by withholding water under the R5 stage due to inoculation with a bioinoculant produced from *Bacillus amyloliquefaciens* and AMF.

Physiological aspects are a valuable tool for studying the impact of drought stress on soybean plants. A higher content of photosynthetic pigment under drought stress indicates that the photosynthetic apparatus is performing well. Our results showed a significant increase in chlorophyll contents in plants inoculated by drought-tolerant bacteria and/or AMF under drought stress (Figure 4A). Such improvements in physiological aspects can lead to more CO_2_ assimilation for photosynthesis. Similarly, several studies have found an improvement in photosynthesis, water status, and antioxidant activity due to AMF inoculation under drought [24,26,27,35,56]. For example, Ashwin et al. [35] observed that dual inoculation with AMF and rhizobia increased the chlorophyll content in drought-stressed soybean plants when compared to uninoculated ones. These findings suggest that microbial inoculation keeps chlorophyll more stable and less susceptible to degradation under drought stress. Organic osmolytes are accumulated in many plants as a response to environmental stresses that cause dehydration [57]. Proline accumulation is a common response to a water deficit in many plants; besides acting as an osmolyte, proline plays an important role in stabilizing sub-cellular structures such as proteins and membranes, as well as free radicals scavenging and cellular redox potential buffering under stress [58]. It is thought that proline can act as a stress marker in addition to its role as an Osmo protectant. It means that plants may accumulate more proline due to a biotic stress or less proline as a result of reduced stress. Our data show that bioinoculant treatments decreased the proline contents under drought when compared to uninoculated treatments (Figure 4B). The low proline accumulation in the inoculated soybean plants indicated that these plants were less exposed to drought and did not require excessive proline accumulation to withstand drought stress. Similarly, several studies have demonstrated the accumulation of proline under drought stress [24,59]. On the contrary, some studies show an increase in proline accumulation due to biofertilizer applications under drought stress [33,60]. Our results suggest the important role of bioinoculants in reducing the redox potential in soybean plants by reducing the proline content.

### 4.4. Drought-Tolerant Bacteria and AMF Improve Nutrient Content in Soybean Plants

The present study showed that the inoculation of soybean plants with bioinoculants leads to an increase in the NPK contents when compared to untreated plants under drought stress (Figure 5). This increase may be attributed to the ability of *Br. japonicum* to fix nitrogen and solubilize P through root nodules and the secretion of organic acids, respectively (Table 4 and Figure 1E). Moreover, *B. subtilis* shows a high ability to solubilize P under drought stress (Figure 2E). In addition, the increase in the mycorrhizal colonization levels under drought stress occurs from the symbiosis between AMF and plant roots, which is involved directly in plant nutrition (Table 2). Similar results were reported by Sheteiwy et al. [34] who found that inoculating soybean plants with *B. amyloliquafaciens* and/or AMF significantly greatly improved key elements such as N, P, and K during drought stress. Similarly, the results of Jabborova et al. [23] show that dual inoculation with *Br. japonicum* USDA 110 and *P. putida* NUU8 significantly increased the NPK contents in soybeans compared to the control under drought conditions. Furthermore, Ashwin et al. [35] found an increase in the macro and micronutrient uptake by drought-stressed soybean plants, specifically N, P, and K, which are crucial for plant growth to tolerate drought stress, and Mg, which is required for chlorophyll formation. Our findings support the use of bioinoculants to increase nutrient uptake during drought stress.

### 4.5. Drought-Tolerant Bacteria and AMF Improve Nodulation and Yield Traits in Soybean Plants

The current results show that inoculation with bioinoculants increased nodulation and yield traits in soybean plants (Table 4). Our findings reveal that bioinoculant applications resulted in increased nodulation, whereas the control had poor root nodulation. This may be due to the low density of native N_2_ fixing symbiotic rhizobium and the low content of soil P [61]. In addition, the results showed that the mixture application showed higher nodule numbers as compared to the individual and dual applications. Similarly, Ngosong et al. [62] found that the combination of PGPB and AMF resulted in a higher number of root nodules when compared to individual applications. Also, Ashwin et al. [35] found that dual inoculation with rhizobia and AMF increased the number and weight of soybean nodules. On the contrary, Sheteiwy et al. [24] found a higher nodule number due to the individual inoculation with *Br. japonicum* when compared to AMF alone or the dual treatment. This may be very dependent on the type of microorganisms that are mixed.

Exposure to drought stress resulted in a significant reduction of yield traits, e.g., pod number, 100 seeds’ weight, and grain yield (Table 4). The reduction in the soybean yield could be attributed to the disruption of carboxylation and the remobilization of photosynthetic products during reproductive growth stages, which in turn leads to seed abortion, reduced seed numbers, and a lower individual seed weight [34,63]. However, our results show that the bioinoculant applications improve yield traits under drought stress (Table 4). This might be due to the ability of drought-tolerant bacteria and AMF to enhance soybean growth, the physiological status, and nutrient contents in soybean plants, which in turn resulted in a high yield and its attributes. Previously, Sheteiwy et al. [24] reported that the use of *Br. japonicum* and AMF had a significant effect on the grain yield of soybean as compared to control plants. Likewise, Ashwin et al. [35] found an increase in the yield attributes of soybean plant under drought stress due to dual inoculation with rhizobia and AMF. Sheteiwy et al. [34] found a significant effect on the pod number and seed weight of soybean plants as affected by the inoculation of *B. amyloliquafaciens* and/or AMF under drought stress. Igiehon et al. [64] found that inoculating with *Rhizobium* spp. and mycorrhizal consortium enhanced the yield and seed size of soybean plants under drought stress.

## 5. Conclusions

Drought stress can reduce soybean growth at critical stages and result in the loss of productivity. Several techniques have been developed to alleviate the adverse effects of drought on soybeans, but these have some potential negative effects and, in addition, are expensive; thus it is necessary to use a cost-effective and environmentally friendly strategy such as microbial inoculation. Hence, in this study, we isolated drought-tolerant bacterial isolates which could help soybean plants withstand drought stress. These bacteria showed a high ability to produce growth-promoting substances under drought stress in vitro, such as indole acetic acid (IAA), gibberellic acid (GA), proline, exopolysaccharides (EPS), and P-solubilized. In addition, understanding how these isolated bacteria along with AMF could mitigate drought stress in soybean plants at critical growth stages is worthwhile. Our results showed that inoculation with drought-tolerant bacteria along with AMF improved the growth traits, physiological indices, nutrient content, and yield of soybean plants under drought stress. As well as the bacterial counts, mycorrhizal colonization indices, and activities of soil enzymes (dehydrogenase and phosphatase), there were also increases in soybean rhizosphere under drought stress. Taken together, the inoculation of *Br. japonicum* PP236808, *B. subtilis* PP250150, and AMF strains in a mixture could help farmers mitigate drought stress on soybean plants while increasing growth and productivity. To understand further the microorganisms–plant–soil interactions under drought stress, underlying signaling and molecular studies are required.

## Figures and Tables

**Table 1:** Growth of drought-tolerant bacterial isolates under drought stress conditions.

*Bradyrhizobium* spp.		Rhizobacteria	
No. of Isolate	OD at 600 nm	No. of Isolate	OD at 600 nm
1 DTB	0.560 ± 0.174 bc	1 DTR	0.373 ± 0.082 c–h
2 DTB	0.610 ± 0.039 bc	2 DTR	0.449 ± 0.120 a–e
3 DTB	0.965 ± 0.026 a	3 DTR	0.531 ± 0.072 a–d
4 DTB	0.930 ± 0.021 a	4 DTR	0.111 ± 0.046 hi
5 DTB	1.000 ± 0.036 a	5 DTR	0.070 ± 0.026 i
6 DTB	0.973 ± 0.028 a	6 DTR	0.072 ± 0.029 i
7 DTB	0.971 ± 0.052 a	7 DTR	0.080 ± 0.102 i
8 DTB	0.183 ± 0.146 e–g	8 DTR	0.086 ± 0.044 i
9 DTB	0.251 ± 0.117 ef	9 DTR	0.154 ± 0.040 g–i
10 DTB	0.169 ± 0.024 e–g	10 DTR	0.117 ± 0.019 hi
11 DTB	0.444 ± 0.043 cd	11 DTR	0.061 ± 0.044 i
12 DTB	0.452 ± 0.084 cd	12 DTR	0.076 ± 0.074 i
13 DTB	0.606 ± 0.064 bc	13 DTR	0.044 ± 0.030 i
14 DTB	0.568 ± 0.123 bc	14 DTR	0.173 ± 0.053 f–i
15 DTB	0.617 ± 0.110 bc	15 DTR	0.065 ± 0.039 i
16 DTB	0.466 ± 0.063 cd	16 DTR	0.262 ± 0.107 e–i
17 DTB	0.477 ± 0.027 cd	17 DTR	0.409 ± 0.135 b–g
18 DTB	0.177 ± 0.139 e–g	18 DTR	0.691 ± 0.509 a
19 DTB	0.185 ± 0.045 e–g	19 DTR	0.256 ± 0.088 e–i
20 DTB	0.218 ± 0.093 e–g	20 DTR	0.643 ± 0.171 ab
21 DTB	0.054 ± 0.053 g	21 DTR	0.161 ± 0.016 g–i
22 DTB	0.453 ± 0.109 cd	22 DTR	0.211 ± 0.068 e–i
23 DTB	0.101 ± 0.038 fg	23 DTR	0.320 ± 0.355 d–i
24 DTB	0.675 ± 0.261 b	24 DTR	0.435 ± 0.067 a–f
25 DTB	0.347 ± 0.070 de	25 DTR	0.189 ± 0.046 e–i
26 DTB	0.558 ± 0.148 bc	26 DTR	0.245 ± 0.060 e–i
27 DTB	0.052 ± 0.024 g	27 DTR	0.211 ± 0.015 e–i
28 DTB	0.465 ± 0.172 cd	28 DTR	0.222 ± 0.133 e–i
29 DTB	0.458 ± 0.099 cd	29 DTR	0.584 ± 0.075 a–c
30 DTB	0.136 ± 0.024 fg	30 DTR	0.643 ± 0.170 ab

Data are means ± SD; different letters within the same column indicate significant differences between means according to Duncan’s multiple-range test at *p* ≤ 0.05. DTB, Drought-tolerant bradyrhizobia; DTR, Drought-tolerant rhizobacteria.

**Table 2:** Bacterial counts (Log (cfu g ^−1^ dry soil)) in root rhizosphere and mycorrhizal colonization levels (%) in the roots of soybean treated with bioinoculants and their combinations under drought stress.

Treatments		Bacterial Counts		Mycorrhizal Colonization %		
		Total	P-Dissolvers	F	M	A
CK	100% NPK	7.062 + 0.054 f	5.911 + 0.031 m	–	–	–
	50% NPK	6.018 + 0.023 h	5.698 + 0.044 n	–	–	–
	Bradyrhiz. + 50% NPK	8.133 + 0.154 e	6.088 + 0.105 g–j	–	–	–
	*B. subtilis* + 50% NPK	8.223 + 0.162 de	6.093 + 0.127 g–i	–	–	–
	AMF + 50% NPK	8.380 + 0.598 a–e	5.994 + 0.023 i–l	89.91 ± 1.10 c	51.00 ± 2.13 a	43.80 ± 2.57 a
	Bradyrhiz.+ *B. subtilis* + 50% NPK	8.319 + 0.047 b–e	6.193 + 0.089 e–g	–	–	–
	Bradyrhiz. + AMF + 50% NPK	8.324 + 0.082 b–e	6.096 + 0.149 g–i	92.33 ± 1.72 b	41.09 ± 1.90 c	35.36 ± 2.44 c
	*B. subtilis* +AMF + 50% NPK	8.261 + 0.051 c–e	6.019 + 0.053 h–l	90.62 ± 1.28 bc	40.20 ± 0.40 c	36.16 ± 1.68 c
	Mixture + 50% NPK	8.467 + 0.004 a–d	6.108 + 0.056 gh	95.67 ± 2.56 a	46.64 ± 1.13 b	38.16 ± 2.73 b
D1	100% NPK	7.248 + 0.015 f	6.054 + 0.006 h–k	–	–	–
	50% NPK	6.259 + 0.048 g	5.982 + 0.016 j–m	–	–	–
	Bradyrhiz. + 50% NPK	8.379 + 0.043 a–e	6.267 + 0.024 b–e	–	–	–
	*B. subtilis* + 50% NPK	8.49 + 0.022 a–d	6.271 + 0.014 b–e	–	–	–
	AMF + 50% NPK	8.479 + 0.124 a–d	6.175 + 0.029 e–g	71.43 ± 0.99 h	32.54 ± 0.79 hj	24.80 ± 0.41 ef
	Bradyrhiz.+ *B. subtilis* + 50% NPK	8.560 + 0.037 ab	6.322 + 0.021 a–d	–	–	–
	Bradyrhiz. + AMF + 50% NPK	8.531 + 0.017 ab	6.355 + 0.029 a–c	76.92 ± 2.70 f	30.14 ± 0.24 j	23.46 ± 2.58 f
	*B. subtilis* +AMF + 50% NPK	8.505 + 0.024 a–c	6.372 + 0.015 ab	73.33 ± 2.14 gh	31.45 ± 0.20 i	21.41 ± 0.20 g
	Mixture + 50% NPK	8.611 + 0.028 a	6.391 + 0.012 a	81.82 ± 3.18 e	36.77 ± 0.58 e	26.44 ± 1.35 de
D2	100% NPK	7.138 + 0.033 f	5.954 + 0.024 k–m	–	–	–
	50% NPK	6.069 + 0.052 gh	5.883 + 0.018 m	–	–	–
	Bradyrhiz. + 50% NPK	8.315 + 0.042 b–e	6.222 + 0.007 d–f	–	–	–
	*B. subtilis* + 50% NPK	8.373 + 0.103 a–e	6.117 + 0.129 f–h	–	–	–
	AMF + 50% NPK	8.24 + 0.124 c–e	6.014 + 0.024 h–l	75.00 ± 1.83 fg	35.54 ± 1.32 ef	26.57 ± 0.19 de
	Bradyrhiz.+ *B. subtilis* + 50% NPK	8.428 + 0.081 a–d	6.258 + 0.047 c–e	–	–	–
	Bradyrhiz. + AMF + 50% NPK	8.398 + 0.081 a–d	6.278 + 0.023 e–b	82.91 ± 2.16 e	34.54 ± 1.30 fg	26.91 ± 1.21 d
	*B. subtilis* +AMF + 50% NPK	8.397 + 0.021 a–d	6.22 + 0.053 d–f	83.33 ± 1.74 e	33.55 ± 0.50 gh	23.05 ± 0.63 fg
	Mixture + 50% NPK	8.471 + 0.078 a–d	6.258 + 0.025 c–e	85.92 ± 0.45 d	38.67 ± 1.01 d	28.50 ± 2.04 d
Fertilization		***	***	***	***	***
Drought		***	***	***	***	***
Fertilization × Drought		***	***	***	***	***

Data are means ± SD (n = 3); different letters within the same column indicate significant differences between means according to Duncan’s multiple-range test at *p* ≤ 0.05; *** denote significant differences at *p* ≤ 0.001, among the studied factors. CK, Check; D1, withholding irrigation at R3 stage; D2, withholding irrigation at R5 stage; AMF, Arbuscular mycorrhizal fungi; F%, the frequency of root colonization; M%, the intensity of cortical colonization; A%, arbuscular frequency in roots.

**Table 3:** Effect of bioinoculants and their combinations on morphological traits and biomass of soybean plants under drought stress.

Treatments		Shoot Length (cm)	Root Length (cm)	Leaf Area (cm^2^)	Dry Weight (g/Plant)
CK	100% NPK	114.43 ± 1.33 e	29.57 ± 1.55 c–f	66.13 ± 4.50 d–f	60.67 ± 1.64 de
	50% NPK	100.27 ± 1.05 fg	19.97 ± 1.24 jk	45.40 ± 3.10 gh	52.82 ± 2.17 f–h
	Bradyrhiz. + 50% NPK	115.60 ± 4.57 e	32.17 ± 3.00 b–d	88.41 ± 9.65 c	59.97 ± 6.07 de
	*B. subtilis* + 50% NPK	122.27 ± 1.01 d	31.27 ± 1.95 b–e	62.85 ± 2.18 ef	57.91 ± 3.08 ef
	AMF + 50% NPK	139.97 ± 4.93 b	35.00 ± 1.51 b	97.00 ± 5.71 b	78.42 ± 2.57 a
	Bradyrhiz.+ *B. subtilis* + 50% NPK	122.63 ± 8.28 d	30.10 ± 1.57 c–f	66.30 ± 2.65 d–f	59.47 ± 5.16 de
	Bradyrhiz. + AMF + 50% NPK	133.40 ± 2.36 c	31.27 ± 2.97 b–e	61.91 ± 3.03 f	65.93 ± 3.67 bc
	*B. subtilis* +AMF + 50% NPK	152.83 ± 4.65 a	44.37 ± 1.07 a	104.33 ± 5.13 b	74.36 ± 5.25 a
	Mixture + 50% NPK	152.30 ± 0.95 a	41.60 ± 2.44 a	113.00 ± 8.19 a	78.63 ± 1.89 a
D1	100% NPK	96.01 ± 5.12 g	15.87 ± 0.49 k	40.13 ± 0.12 gh	47.26 ± 1.00 ij
	50% NPK	71.20 ± 0.90 h	11.57 ± 1.68 l	16.57 ± 4.56 j	39.95 ± 1.35 k
	Bradyrhiz. + 50% NPK	101.53 ± 4.85 fg	15.97 ± 0.76 k	38.47 ± 5.02 h	49.67 ± 1.83 hi
	*B. subtilis* + 50% NPK	100.90 ± 4.97 fg	15.93 ± 0.50 k	48.13 ± 1.01 g	50.26 ± 1.02 hi
	AMF + 50% NPK	100.30 ± 0.20 fg	16.67 ± 1.19 k	84.17 ± 4.38 c	54.43 ± 1.11 f–h
	Bradyrhiz.+ *B. subtilis* + 50% NPK	102.83 ± 3.29 fg	19.67 ± 3.13 jk	41.53 ± 2.16 gh	49.99 ± 1.41 hi
	Bradyrhiz. + AMF + 50% NPK	112.40 ± 1.05 e	23.80 ± 0.70 g–j	45.13 ± 1.53 gh	52.93 ± 0.34 f–h
	*B. subtilis* +AMF + 50% NPK	112.67 ± 1.68 e	30.93 ± 8.55 b–e	70.93 ± 1.21 de	50.98 ± 5.34 hi
	Mixture + 50% NPK	112.23 ± 3.40 e	30.20 ± 0.46 c–f	72.30 ± 4.56 d	61.33 ± 0.02 c–e
D2	100% NPK	111.57 ± 1.108 e	22.13 ± 0.74 ij	42.33 ± 6.60 gh	51.94 ± 3.31 hi
	50% NPK	98.20 ± 0.85 fg	16.07 ± 0.42 k	29.63 ± 8.30 i	43.62 ± 1.88 jk
	Bradyrhiz. + 50% NPK	113.57 ± 5.82 e	23.33 ± 0.06 h–j	61.33 ± 1.53 f	57.29 ± 1.14 e–g
	*B. subtilis* + 50% NPK	114.27 ± 0.90 e	27.23 ± 2.89 e–h	46.00 ± 3.48 gh	52.27 ± 1.53 g–i
	AMF + 50% NPK	124.93 ± 2.00 d	26.07 ± 3.66 f–i	84.74 ± 5.00 c	66.63 ± 3.68 b
	Bradyrhiz.+ *B. subtilis* + 50% NPK	114.90 ± 1.47 e	23.73 ± 0.32 g–j	42.75 ± 2.15 gh	52.97 ± 1.27 f–h
	Bradyrhiz. + AMF + 50% NPK	121.90 ± 7.02 d	27.90 ± 2.07 d–g	47.33 ± 2.31 gh	61.30 ± 1.16 c–e
	*B. subtilis* +AMF + 50% NPK	131.47 ± 1.18 c	41.10 ± 0.69 a	80.43 ± 9.87 c	53.22 ± 2.08 f–h
	Mixture + 50% NPK	134.83 ± 3.56 bc	33.60 ± 2.20 bc	81.60 ± 1.21 c	64.53 ± 2.05 b–d
Fertilization		***	***	***	***
Drought		***	***	***	***
Fertilization × Drought		***	***	***	***

Data are means ± SD; different letters within the same column indicate significant differences between means according to Duncan’s multiple-range test at *p* ≤ 0.05; *** denote significant differences at *p* ≤ 0.001, among the studied factors. CK, Check; D1, withholding irrigation at R3 stage; D2, withholding irrigation at R5 stage; AMF, Arbuscular mycorrhizal fungi.

**Table 4:** Effect of bioinoculants and their combinations on nodulation and yield traits of soybean plant under drought stress.

Treatments		Nodules/Plant	Pods/Plant	100 Seeds Weight (g)	Grain Yield (t/ha)
CK	100% NPK	12.67 ± 2.08 jk	52.67 ± 2.89 de	15.17 ± 0.39 cd	2.06 ± 0.10 c–f
	50% NPK	9.67 ± 0.58 l–o	43.33 ± 2.89 e–h	10.81 ± 0.68 gh	1.23 ± 0.12 k–m
	Bradyrhiz. + 50% NPK	22.67 ± 2.08 a–c	54.00 ± 4.00 d	15.71 ± 0.51 c	2.41 ± 0.45 ab
	*B. subtilis* + 50% NPK	13.67 ± 0.58 ij	82.33 ± 3.06 ab	15.80 ± 0.50 c	2.04 ± 0.05 d–g
	AMF + 50% NPK	12.00 ± 1.00 j–l	63.67 ± 9.02 c	17.57 ± 0.46 ab	2.10 ± 0.04 b–f
	Bradyrhiz.+ *B. subtilis* + 50% NPK	20.33 ± 0.58 c–e	63.33 ± 9.07 c	15.16 ± 0.93 cd	2.37 ± 0.23 a–d
	Bradyrhiz. + AMF + 50% NPK	22.67 ± 1.53 a–c	76.00 ± 6.08 ab	16.48 ± 0.72 a–c	2.16 ± 0.04 a–e
	*B. subtilis* +AMF + 50% NPK	15.67 ± 0.58 hi	72.67 ± 15.53 b	16.11 ± 1.53 bc	2.38 ± 0.33 a–c
	Mixture + 50% NPK	23.67 ± 1.15 a	83.67 ± 4.62 ab	17.81 ± 0.44 a	2.46 ± 0.41 a
D1	100% NPK	7.67 ± 1.15 op	38.67 ± 0.58 gh	10.92 ± 0.67 g	1.12 ± 0.20 l–n
	50% NPK	5.33 ± 0.58 p	28.00 ± 2.65 i	9.31 ± 0.20 h	0.85 ± 0.17 n
	Bradyrhiz. + 50% NPK	18.33 ± 1.53 e–g	39.00 ± 1.00 f–h	10.24 ± 0.50 gh	1.22 ± 0.15 k–m
	*B. subtilis* + 50% NPK	8.33 ± 1.15 no	37.00 ± 4.36 h	10.18 ± 0.67 gh	1.17 ± 0.03 l–n
	AMF + 50% NPK	10.00 ± 1.00 l–o	35.67 ± 4.04 hi	11.31 ± 0.19 fg	1.13 ± 0.23 l–n
	Bradyrhiz.+ *B. subtilis* + 50% NPK	17.33 ± 3.79 f–h	40.00 ± 3.61 f–h	11.26 ± 1.08 fg	1.07 ± 0.04 mn
	Bradyrhiz. + AMF + 50% NPK	11.33 ± 1.15 j–m	38.33 ± 1.15 gh	10.34 ± 0.95 gh	1.71 ± 0.18 g–j
	*B. subtilis* +AMF + 50% NPK	9.33 ± 0.58 m–o	37.33 ± 3.21 h	11.34 ± 1.57 fg	1.41 ± 0.05 j–l
	Mixture + 50% NPK	17.00 ± 1.00 gh	44.00 ± 1.00 e–h	11.20 ± 1.01 fg	1.53 ± 0.01 i–k
D2	100% NPK	10.00 ± 1.00 l–o	44.67 ± 4.51 d–h	13.10 ± 0.70 e	1.63 ± 0.11 h–j
	50% NPK	7.67 ± 0.58 op	38.33 ± 1.15 gh	10.34 ± 0.82 gh	1.02 ± 0.03 mn
	Bradyrhiz. + 50% NPK	23.33 ± 1.53 ab	45.67 ± 1.53 d–h	14.07 ± 0.78 de	1.93 ± 0.07 e–h
	*B. subtilis* + 50% NPK	10.33 ± 0.58 k–n	51.00 ± 5.29 de	12.59 ± 1.22 ef	1.63 ± 0.02 h–j
	AMF + 50% NPK	10.67 ± 0.58 k–n	45.33 ± 5.13 d–h	13.54 ± 0.38 e	1.65 ± 0.30 h–j
	Bradyrhiz.+ *B. subtilis* + 50% NPK	21.00 ± 1.73 b–d	48.67 ± 1.15 d–f	12.96 ± 0.29 e	1.42 ± 0.18 j–l
	Bradyrhiz. + AMF + 50% NPK	19.33 ± 2.08 d–g	48.33 ± 1.53 d–g	13.68 ± 1.52 de	1.81 ± 0.09 f–i
	*B. subtilis* +AMF + 50% NPK	11.67 ± 1.53 j–m	44.33 ± 7.51 d–h	12.66 ± 0.92 ef	1.71 ± 0.10 g–j
	Mixture + 50% NPK	19.67 ± 0.58 d–f	52.33 ± 1.15 de	13.67 ± 1.01 de	2.04 ± 0.06 d–g
Fertilization		***	***	***	***
Drought		***	***	***	***
Fertilization × Drought		***	***	***	***

Data are means ± SD; different letters within the same column indicate significant differences between means according to Duncan’s multiple-range test at *p* ≤ 0.05; *** denote significant differences at *p* ≤ 0.001, among the studied factors. CK, Check; D1, withholding irrigation at R3 stage; D2, withholding irrigation at R5 stage; AMF, Arbuscular mycorrhizal fungi.

## Data Availability

The raw data supporting the conclusions of this article will be made available by the authors on request.

## Supplementary Materials

The following supporting information can be downloaded at: https://www.mdpi.com/article/10.3390/microorganisms12061123/s1, Table S1: Meteorological data of temperature (°C), relative humidity (%), and rainfall amount (mm) during 2023 growing season; Table S2: Physicochemical and biological properties of soil used in 2023 growing season; Figure S1: Phylogenetic trees of (A) *Bredyrhizobium japonicum* PP236808 (B) *Bacillus subtilis* PP250150.
