# Supplementary material for: Drought-Tolerant Bacteria and Arbuscular Mycorrhizal Fungi Mitigate the Detrimental Effects of Drought Stress Induced by Withholding Irrigation at Critical Growth Stages of Soybean (Glycine max, L.)
Source: Microorganisms. 2024 May 31;12(6):1123. doi: 10.3390/microorganisms12061123 (PMC11205826; doi:10.3390/microorganisms12061123)
Supplement: Supplementary file 1 [file microorganisms-12-01123-s001.zip › microorganisms-2979528-supplementary.pdf]

Drought-Tolerant Bacteria and Arbuscular Mycorrhizal Fungi Mitigate the Detrimental Effects of Drought Stress Induced by Withholding Irrigation at Critical Growth Stages of Soybean (*Glycine max*, L.)

Aya Ahmed Nader, Fathi I. A. Hauka, Aida H. Afify and Ahmed M. El-Sawah *

Department of Agricultural Microbiology, Faculty of Agriculture, Mansoura University, Mansoura 35516, Egypt

* Correspondence: ahmedelsawah89@mans.edu.eg

Table S1. Meteorological data of temperature (°C), relative humidity (%), and rainfall amount (mm) during 2023 growing season.

Year<br>Month	2023			
	Temperature (°C)		Humidity (%)	Rainfall (mm)
	Max	Min		
May	33	17	53	1.30
June	37	21	52	0.80
July	40	23	51	0.00
August	38	22	59	0.00
September	37	21	60	0.00
October	31	20	60	0.18
November	20	17	60	10.03

Table S2. Physicochemical and biological properties of soil used in 2023 growing season.

Property	Value
**Particle size distribution (%)**	
Sand	48.23
Silt	30.64
Clay	21.13
**Chemical analysis**	
pH	8.16
EC	0.80
**Cations (meq/100g)**	
Ca++	0.62
Mg++	1.55
Na+	0.83
K+	0.04
**Anions (meq/100g)**	
CO_3^-	0.00
HCO_3^-	0.08
Cl^-	0.52
**Available nutrients (mg/kg)**	
N	14.76
P	13.41
K	355.04
**Bacterial count**	
TBC	5.917
PDC	4.789

pH (1:2.5); EC (electrical conductivity dsm^{-1}); TBC total bacterial count log (cfu g^{-1} dry soil) and PDC phosphate dissolvers count log (cfu g^{-1} dry soil)

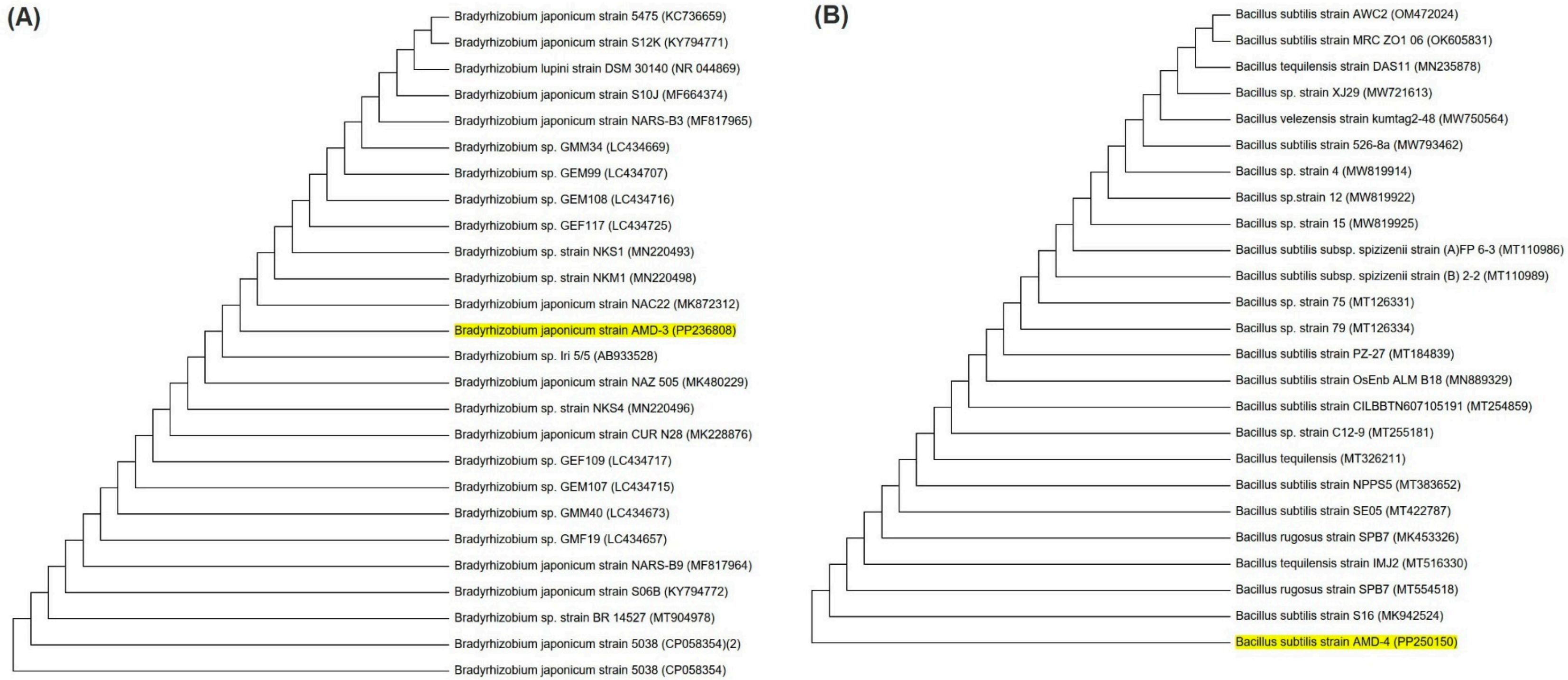

Figure S1. Phylogenetic trees of (A) *Bredyrhizobium japonicum* PP236808 (B) *Bacillus subtilis* PP250150
